# MicroRNA Gene Polymorphisms and Environmental Factors Increase Patient Susceptibility to Hepatocellular Carcinoma

**DOI:** 10.1371/journal.pone.0089930

**Published:** 2014-02-26

**Authors:** Yin-Hung Chu, Ming-Ju Hsieh, Hui-Ling Chiou, Yi-Sheng Liou, Chen-Chieh Yang, Shun-Fa Yang, Wu-Hsien Kuo

**Affiliations:** 1 Institute of Medicine, Chung Shan Medical University, Taichung, Taiwan; 2 Cancer Research Center, Changhua Christian Hospital, Changhua, Taiwan; 3 School of Medical Laboratory and Biotechnology, Chung Shan Medical University, Taichung, Taiwan; 4 Department of Family Medicine, Taichung Veterans General Hospital, Taichung, Taiwan; 5 Department of Public Health, National Defense Medical Center, Taipei, Taiwan; 6 Division of Gastroenterology, Department of Internal Medicine, Mennonite Christian Hospital, Hualien, Taiwan; 7 Department of Medical Research, Chung Shan Medical University Hospital, Taichung, Taiwan; 8 Department of Medicine, Armed-Force Taichung General Hospital, Taichung, Taiwan; Centro di Riferimento Oncologico, IRCCS National Cancer Institute, Italy

## Abstract

**Background:**

Micro RNAs (miRNAs) are small RNA fragments that naturally exist in the human body. Through various physiological mechanisms, miRNAs can generate different functions for regulating RNA protein levels and balancing abnormalities. Abnormal miRNA expression has been reported to be highly related to several diseases and cancers. Single-nucleotide polymorphisms (SNPs) in miRNAs have been reported to increase patient susceptibility and affect patient prognosis and survival. We adopted a case-control research design to verify the relationship between miRNAs and hepatocellular carcinoma.

**Methodology/Principal Findings:**

A total of 525 subjects, including 377 controls and 188 hepatocellular carcinoma patients, were selected. Polymerase chain reaction-restriction fragment length polymorphism (PCR-RFLP) and real-time PCR were used to analyze miRNA146a (rs2910164), miRNA149 (rs2292832), miRNA196 (rs11614913), and miRNA499 (rs3746444) genetic polymorphisms between the control group and the case group. The results indicate that people who carry the rs3746444 CT or CC genotypes may have a significantly increased susceptibility to hepatocellular carcinoma (adjusted odds ratio [AOR] = 2.84, 95% confidence interval [CI] = 1.88–4.30). In addition, when combined with environmental risk factors, such as smoking and alcohol consumption, interaction effects were observed between gene polymorphisms and environmental factors (odds ratio [OR] = 4.69, 95% CI = 2.52–8.70; AOR = 3.38, 95% CI = 1.68–6.80).

**Conclusions:**

These results suggest that a significant association exists between miRNA499 SNPs and hepatocellular carcinoma. Gene-environment interactions of miRNA499 polymorphisms, smoking, and alcohol consumption might alter hepatocellular carcinoma susceptibility.

## Introduction

Hepatocellular carcinoma (HCC) is one of the most common malignancies worldwide and the second leading cause of cancer-related death in Taiwan. In addition to common risk factors such as hepatitis B and hepatitis C, recent studies have shown that gene mutations may influence the inflammation mechanism that contributes to cell differentiation, proliferation, and apoptosis and connects several diseases and cancers [1]–[3]. Genotypes and mutations on hepatitis were associated to HCC, such as genotype C HBV increased the risk of cirrhosis and HCC, and genotype B HBV is associated with HCC in the patients under 50 years old [4]. Also, viral mutations on the promoter region and preS region are significantly increased the risk of HCC. The variances of genotype, mutations and viral load can be used as a prediction of HCC [5].

Micro RNAs (miRNAs) are small RNA fragments typically composed of only 20–22 nucleotides. MiRNAs can target specific mRNAs and negatively regulate their translational efficiency and stability to control the downstream cellular process including proliferation, differentiation, and survival [6], [7]. According to the various functions of miRNAs, some studies have associated miRNAs with the immune system and inflammatory diseases [8], [9]. Moreover, based on the functions that regulate cell differentiation, proliferation, and survival, several studies have identified a relationship between miRNAs and cancer [10]–[14].

Single-nucleotide polymorphisms (SNPs) are variations of DNA sequences that occur when nucleotides (A, T, C, or G) are changed by at least 1% in certain populations. Previous epidemiologic studies have shown that genetic variations in miRNAs are associated with various diseases and cancers [15]–[18]. Several studies have provided evidence that SNPs in miRNAs may be related to patients’ clinical prognosis [19]–[22], and high expression of miRNA causes superior survival. According to the various functions, low expression of miRNA can yield opposite results [23], [24].

Based on the aforementioned reasons, we adopted a case-control research design and 4 pre-miRNA SNPs associated with cancer, namely, has-mir-146a (rs2910164), has-mir-149 (rs2292832), has-mir-196a2 (rs11614913), and has-mir-499 (rs3746444) [25]–[29]. We also considered the environmental risk factors for hepatocellular carcinoma such as smoking status and alcohol consumption. Furthermore, the clinical stage and laboratory status were combined to define the relationship between SNPs and patient susceptibility to hepatocellular carcinoma.

## Materials and Methods

### Subjects Selection

This study recruited 188 HCC patients at the Chung Shan Medical University Hospital, Taiwan. The diagnosis of HCC was made according to the criteria specified in the national guidelines for HCC. During the same study period, 337 ethnic group-matched individuals were enrolled as the controls that entered the physical examination at the same hospital. These control groups had neither self-reported history of cancer of any sites. As for cases and controls, exposure information, including tobacco use (smoker vs. non-smoker) and alcohol consumption (current heavy drinker, defined by CDC as consuming an average of more than 2 drinks per day vs. not current heavy drinker), were all obtained from questionnaires. We also collected the laboratory status such as α-Fetoprotein, AST, ALT and AST/ALT ratio for the further analysis. Before commencing the study, approval was obtained from the Institutional Review Board of Chung Shan Medical University Hospital and informed written consent was obtained from each individual.

### DNA Extraction

We collected the whole blood samples from healthy controls and hepatocellular carcinoma patients with tubes containing EDTA, after centrifuged and stored at −20°C. The venous blood from each subject was drawn into Vacutainer tubes containing EDTA and stored at 4°C. Genomic DNA was extracted by QIAamp DNA blood mini kits (Qiagen, Valencia, USA) according to the manufacture’s instructions and the DNA was dissolved in TE buffer [10 mM Tris (PH 7.8), 1 mM EDTA] and then quantitated by a measurement of OD260. Final DNA preparation was stored at −20°C and used as templates for the following experiments [30].

### Polymerase Chain Reaction-restriction Fragment Length Polymorphism (PCR-RFLP)

The SNP of miRNAs gene rs2910164, rs11614913, and rs3746444 polymorphisms were determined by PCR-RFLP assay [31]. Amplify The has-mir-146a rs2910164 genotype were used the forward primer 5′- CATGGGTTGTGTCAGTGTCAGAGCT-3′ and the reverse primer 5′- TGCCTTC TGTCTCCAGTCTTCCAA-3′; the has-mir-196a2 rs 11614913 genotype were used forward primer 5′- CCCCTTCCCTTCTCCTCCAGATA-3′ and reverse primer 5′- CGAAAACCGACTGATGTAACTCCG-3′ and the has-mir-499 rs3746444 genotype were used forward primer 5′-CAAAGTCTTCACTTCCCTGCCA-3′ and reverse primer 5′-GATGTTTAACTCCTCTCCACGTGATC-3′. PCR was performed with total 10 µl volume with 100 ng DNA template, 1.0 µl of 10X PCR buffer (Invitrogen, Carslbad, CA, USA), 0.25 U of Taq DNA polymerase (Invitrogen), 0.2 mM dNTPs (Promega, Madison, WI, USA) and 200 nM of each primer (MDBio Inc. Taipei, Taiwan). The PCR conditions were 5 min at 94°C followed by 35 cycles of 30 sec at 94°C, 30 sec at 58°C for hsa-mir-146a rs2910164, 30 sec at 63°C for has-mir-196a2 rs11614913, and 30 sec at 67°C for hsa-mir-499 rs3746444, and 1 min at 72°C, and final step at 72°C for 10 min to allow a complete extension of all PCR fragments. PCR products of hsa-mir-146a rs2910164 gene polymorphism were subjected *SacI* to enzymatic digestion by incubation for 4 hr at 37°C and then submitted to electrophoresis in 2% agarose gels; *MspI* for hsa-mir-196a2 rs2910164 and *BclI* for hsa-mir-499 rs3746444.

### Real-time PCR

MiRNA149 rs2292832 gene polymorphism was assessed using an ABI StepOne™ Real-Time PCR System (Applied Biosystems), SDS v3.0 software (Applied Biosystems), and the TaqMan assay. The final volume for each reaction mixture was 5 µL, containing 2.5 µL TaqMan Genotyping Master Mix, 0.125 µL TaqMan probes mix, and 10 ng genomic DNA. The reaction conditions included an initial denaturation step at 95°C for 10 min followed by 40 cycles at 95°C for 15 sec and 60°C for 1 min. For each assay, appropriate controls (nontemplate and known genotype) were included in each typing run to monitor reagent contamination and as a quality control. To validate results from real-time PCR, around 5% of assays were repeated and several cases of each genotype were confirmed by the DNA sequence analysis [32].

### Statistical Analysis

Hardy–Weinberg equilibrium was assessed using a chi-square goodness-of-fit test for biallelic markers. The distributions of demographic characteristics and genotype frequencies between cases and controls in different genotypes were analyzed by Chi-square test, and Fisher’s exact test were using at small sample size was present in some categories of variables. The Student’s T-test was using to estimate laboratory status between the two groups. The odds ratios (ORs) and their 95% confidence intervals (CIs) of the association between genotype frequencies and hepatocellular carcinoma were estimated by multiple logistic regression models, also controlling for covariates. The *p* value of less than 0.05 was considered significant. The data were analyzed on SPSS 12.0 statistical software.

## Results

### Study Population

The demographic characteristics of the 2 groups, including age, sex, smoking status, and alcohol consumption, are shown in Table 1. A significant difference in smoking status was observed between the 2 groups (*p = *0.03).

**Table 1:** Demographic characteristics of controls and patients with hepatocellular carcinoma.

Variable	Control	Case	Total	*p* value
	N = 337 (%)	N = 188 (%)	N = 525 (%)	
Age				
<45	24 (7.12)	10 (5.05)	34 (6.48)	0.06
45–59	210 (40.06)	58 (30.85)	193 (36.76)	
>60	178 (52.82)	120 (63.83)	298 (56.76)	
Gender				
Women	85 (25.22)	52 (27.66)	137 (26.10)	0.54
Men	252 (74.78)	136 (72.34)	388 (73.90)	
Smoking status				
No	225 (66.77)	108 (57.45)	333 (63.43)	0.03*
Yes	112 (33.23)	80 (42.55)	192 (36.57)	
Alcohol intake				
No	201 (59.64)	120 (63.83)	321 (61.14)	0.35
Yes	136 (40.36)	68 (36.17)	204 (38.86)	
Hepatitis B virus^a^				
Negative	223 (86.77)	108 (57.45)	331 (74.38)	<0.001*
Positive	34 (13.23)	80 (42.55)	114 (25.62)	

*p<0.05 considered statistically significant.

a 80 frequency missing data in control group.

### MiRNA Gene Polymorphisms

The distribution of miRNA gene polymorphisms is described in Table 2. In our recruited control group, the frequencies of miRNA146a rs2910164 (χ^2^ value: 1.92), miRNA196 rs11614913 (χ^2^ value: 0.0005), and miRNA499 rs3746444 (χ^2^ value: 0.98) were in Hardy-Weinberg equilibrium, respectively, except for miRNA149 rs2292832 (χ^2^ value: 59.86). By using a multiple logistic regression model to estimate the adjusted odds ratio (AOR), we observed a significantly higher risk for people who carried the CT (AOR = 2.52, 95% confidence interval [CI] = 1.63–3.86) or CC (AOR = 20.7, 95% CI = 2.60–165.90) genotypes of miRNA499 (rs3746444). Because of the insufficient size of the CC group, the CT and CC genotype samples were combined, producing similar results (AOR = 2.84, 95% CI = 1.88–4.30). In addition, no statistical significance was observed in the other 3 gene polymorphisms (rs2910164, rs2292832, and rs11614913). Similarly, a significantly higher risk for people who carried the CT (AOR = 6.14, 95% confidence interval [CI] = 1.32–28.56) or CC (AOR = 4.75, 95% CI = 1.32–17.01) genotypes of miRNA499 (rs3746444) was observed in the HBV-infection group (Table S1 in File S1).

**Table 2:** Distribution of miRNAs genotypes in healthy controls and hepatocellular carcinoma patients.

Gene	Control	Case	OR	AOR^a^
	N = 337 (%)	N = 188 (%)	(95%CI, *p* value)	(95%CI, *p* value)
miRNA146ars2910164				
CC	141 (41.84)	84 (44.68)	Reference	Reference
CG	146 (43.32)	82 (43.62)	0.94 (0.64–1.38, *p* = 0.763)	0.93 (0.63–1.37, *p* = 0.714)
GG	50 (14.84)	22 (11.70)	0.74 (0.42–1.31, *p* = 0.297)	0.72 (0.40–1.27, *p* = 0.253)
CG/GG	196 (58.16)	104 (55.32)	0.89 (0.62–1.28, *p* = 0.528)	0.83 (0.59–1.18, *p* = 0.300)
miRNA149 rs2292832				
TT	246 (73.00)	139 (73.94)	Reference	Reference
CT	64 (18.99)	36 (19.15)	0.99 (0.63–1.57, *p* = 0.984)	0.99 (0.62–1.58, *p* = 0.982)
CC	27 (8.01)	13 (6.91)	0.85 (0.43–1.71, *p* = 0.651)	0.82 (0.41–1.66, *p* = 0.579)
CT/CC	91 (27.00)	49 (26.06)	0.95 (0.64–1.43, *p* = 0.816)	0.94 (0.63–1.41, *p* = 0.767)
miRNA196 rs11614913				
TT	100 (29.67)	66 (35.11)	Reference	Reference
CT	167 (49.55)	81 (43.09)	0.74 (0.49–1.11, *p* = 0.140)	0.70 (0.46–1.06, *p* = 0.090)
CC	70 (20.77)	41 (21.81)	0.89 (0.54–1.46, *p* = 0.637)	0.93 (0.56–1.54, *p* = 0.785)
CT/CC	237 (70.33)	122 (64.89)	0.78 (0.53–1.14, *p* = 0.200)	0.90 (0.64–1.26, *p* = 0.544)
miRNA499 rs3746444				
TT	281 (83.38)	119 (63.30)	Reference	Reference
CT	55 (16.32)	60 (31.91)	2.58 (1.69–3.94, *p*<0.001)*	2.48 (1.62–3.81, *p*<0.001)*
CC	1 (0.30)	9 (4.79)	21.2 (2.66–169.55, *p* = 0.004)*	22.1 (2.73–178.40, *p* = 0.003)*
CT/CC	56 (16.62)	69 (36.70)	2.91 (1.93–4.40, *p*<0.001)*	2.91 (1.93–4.42, *p*<0.001)*

a AOR adjusted age, sex, smoking status and drinking status.

*p value <0.05.

### Gene Polymorphisms with Clinicopathologic TNM Staging

The distribution of clinical status and miRNA polymorphisms in HCC patients were estimated to clarify the role of miRNA polymorphisms in the clinicopathologic state of HCC patients. No significant association was found between the levels of these HCC clinical pathological markers as well as presence of HBV or HCV, liver cirrhosis and genotypes for any of the RNA SNPs in HCC patients (Table 3 and Table 4). However, the trend of TC+CC genotype of miRNA rs2292832 for distant metastasis risk was higher in the male participants (Table S2 in File S1) while that of female participants was not (Table S3 in File S1).

**Table 3:** Relationship of clinical TNM stages and miRNAs genotypes in liver cancer patients.

Variables	rs2910164			rs2292832			rs11614913			rs3746444		
	CC	CG or GG	*p*value	TT	CT or CC	*p*value	TT	CT or CC	*p*value	TT	CT or CC	*p*value
	N = 84 (%)	N = 104 (%)		N = 139 (%)	N = 49 (%)		N = 66 (%)	N = 122 (%)		N = 119 (%)	N = 69 (%)	
Clinical stage												
Stage I/II	49 (58.33)	66 (63.46)	0.471	85 (61.15)	30 (61.22)	0.992	40 (60.61)	75 (61.48)	0.911	73 (61.34)	42 (60.87)	0.947
Stage III/IV	35 (41.67)	38 (36.54)		54 (38.85)	19 (38.78)		26 (39.39)	47 (38.52)		46 (38.66)	27 (39.13)	
Tumor size												
T1+ T2	50 (59.52)	67 (64.42)	0.496	88 (63.31)	29 (59.18)	0.615	39 (59.09)	78 (63.93)	0.510	75 (63.03)	42 (60.87)	0.775
T3+ T4	34 (40.48)	37 (35.58)		51 (36.69)	20 (40.82)		27 (40.91)	44 (36.07)		44 (36.97)	27 (39.13)	
Lymph node metastasis												
Negative	79 (94.05)	100 (96.15)	0.514^a^	131 (94.24)	48 (97.96)	0.294^a^	62 (93.94)	117 (95.90)	0.554^a^	112 (94.12)	67 (97.10)	0.353^a^
Positive	5 (5.95)	4 (3.85)		8 (5.76)	1 (2.04)		4 (6.06)	5 (4.10)		7 (5.88)	2 (2.90)	
Distant metastasis												
Negative	78 (92.86)	99 (95.19)	0.493	133 (95.68)	44 (89.80)	0.136	62 (93.94)	115 (94.26)	0.922^a^	110 (92.44)	67 (97.10)	0.194^a^
Positive	6 (7.14)	5 (4.81)		6 (4.32)	5 (10.20)		4 (6.06)	7 (5.74)		9 (7.56)	2 (2.90)	

a Use Fisher’s exact test.

**Table 4:** Relationship of clinical statuses and miRNAs genotypes in liver cancer patients.

Variables	rs2910164			rs2292832			rs11614913			rs3746444		
	CC	CG or GG	*p*value	TT	CT or CC	*p*value	TT	CT or CC	*p*value	TT	CT or CC	*p*value
	N = 84 (%)	N = 104 (%)		N = 139 (%)	N = 49 (%)		N = 66 (%)	N = 122 (%)		N = 119 (%)	N = 69 (%)	
HBsAg												
Negative	52 (61.90)	57 (54.81)	0.322	82 (58.99)	27 (55.10)	0.633	33 (50.00)	76 (62.30)	0.102	66 (55.46)	43 (62.32)	0.357
Positive	32 (38.10)	47 (45.19)		57 (41.01)	22 (44.90)		33 (50.00)	46 (37.70)		53 (44.54)	26 (37.68)	
Anti-HCV												
Negative	44 (52.38)	53 (50.96)	0.841	69 (49.64)	28 (57.14)	0.367	34 (51.52)	63 (51.64)	0.983	67 (56.30)	30 (43.48)	0.079
Positive	40 (47.62)	51 (49.04)		70 (50.36)	21 (42.86)		32 (48.48)	59 (48.36)		52 (43.70)	39 (56.52)	
Child-Pugh grade												
A	62 (73.81)	75 (72.12)	0.796	103 (74.10)	34 (69.39)	0.526	46 (69.70)	91 (74.59)	0.475	85 (71.43)	52 (75.36)	0.551
B or C	22 (26.19)	29 (27.88)		36 (25.90)	15 (30.61)		20 (30.30)	31 (25.41)		34 (28.57)	17 (24.64)	
Liver Cirrhosis												
Negative	21 (25.00)	29 (27.88)	0.654	41 (29.50)	9 (18.37)	0.129	13 (19.70)	37 (30.33)	0.113	30 (25.21)	20 (28.99)	0.572
Positive	63 (75.00)	75 (72.12)		98 (70.50)	40 (81.63)		53 (80.30)	85 (69.67)		89 (74.79)	49 (71.01)	

a Use Fisher’s exact test.

### Gene Polymorphisms with Laboratory Status

We associated laboratory status with gene polymorphisms, including α-fetoprotein (ng/mL), AST (IU/L), ALT (IU/L), and AST/ALT ratios, as shown in Table 5. We observed a significantly lower ratio in the patients who carried rs2910164 CG or GG genotypes (*p* = 0.003).

**Table 5:** Relationship of HCC laboratory status and miRNAs genotypes in liver cancer patients.

Variables	α-Fetoprotein	AST	ALT	AST/ALT
	(ng/ml)	(IU/L)	(IU/L)	ratio
miRNA146a rs2910164				
CC	4211.1±18853	192.93±363.25	122.52±171.11	1.73±1.44
CG/GG	4459.9±16050	196.55±389.84	196.84±362.98	1.25±0.70
*p* value	0.922	0.948	0.086	0.003*
miRNA149 rs2292832				
TT	3584.9±16267	198.77±401.56	170.74±320.38	1.42±1.04
CT/CC	6515.6±20006	184.02±300.92	143.48±207.47	1.59±1.33
*p* value	0.309	0.815	0.579	0.359
miRNA196 rs11614913				
TT	3599.4±14256	199.35±416.04	173.52±332.77	1.42±0.74
CT/CC	4754.1±18801	192.54±356.22	158.29±273.48	1.49±1.29
*p* value	0.664	0.906	0.736	0.705
miRNA499 rs3746444				
TT	2871.7±11986	214.91±434,71	167.92±333.43	1.54±1.24
CT/CC	6896.2±23757	160.48±247.88	156.25±214.63	1.34±0.87
*p* value	0.125	0.342	0.794	0.254

*p value <0.05.

### Gene Polymorphism with Environmental Risk Factors

Furthermore, we considered the environmental risk factors of hepatocellular carcinoma such as smoking and alcohol consumption. The risk factors were then combined with miRNA gene polymorphisms and examined to determine whether interactions existed between the 2 factors. The relationship between genotype and smoking status indicated that people who carried rs3746444 CT or CC genotypes and non-smoking habits had an increased odds ratio (OR = 2.34, 95% CI = 1.36–4.00), and those with CT or CC genotypes and smoking habits had the highest odds ratio (OR = 4.69, 95% CI = 2.52–8.70) (Table 6). The result for patients who carried the re3746444 CT or CC genotypes was similar to that observed for patients who consumed alcohol (AOR = 3.44, 95% CI = 1.69–7.00) (Table 7).

**Table 6:** Association of miRNA genotype and smoking status.

Variable	Control	Case	OR	AOR^a^
	N = 337 (%)	N = 188 (%)	(95%CI, *p* value)	(95%CI, *p* value)
miRNA146a rs2910164				
CC and non-smoker	94 (27.89)	50 (26.60)	Reference	Reference
CG or GG and non-smoker	131 (38.87)	58 (30.85)	0.83 (0.53–1.32, *p* = 0.436	0.80 (0.50–1.31, *p* = 0.376)
CC and smoker	47 (13.95)	34 (18.09)	1.36 (0.78–2.38, *p* = 0.280)	1.37 (0.78–2.47, *p* = 0.293)
CG or GG and smoker	65 (19.29)	46 (24.47)	1.33 (0.80–2.22, *p* = 0.273)	1.36 (0.78–2.36, *p* = 0.278)
Test for interaction	?^2^ = 5.102 (3 d.f.), *p* = 0.164			
miRNA149 rs2292832				
TT and non-smoker	164 (48.66)	50 (43.09)	Reference	Reference
CT or CC and non-smoker	61 (18.10)	27 (14.36)	0.90 (0.53–1.52, *p* = 0.682)	0.85 (0.49–1.47, *p* = 0.550)
CT or CC and smoker	82 (24.33)	58 (30.85)	1.43 (0.93–2.20, *p* = 0.101)	1.66 (1.05–2.64, *p* = 0.003)*
CT or CC and smoker	30 (8.90)	114 (11.70)	1.49 (0.81–2.74, *p* = 0.205)	1.88 (0.99–3.59, *p* = 0.054)
Test for interaction	?^2^ = 4.689 (3 d.f.), *p = *0.1960			
miRNA196 rs11614913				
TT and non-smoker	74 (21.96)	34 (18.09)	Reference	Reference
CT or CC and non-smoker	151 (44.81)	74 (39.36)	1.07 (0.65–1.75, *p* = 0.797)	0.99 (0.59–1.66, *p* = 0.962)
CT or CC and smoker	26 (7.72)	32 (17.02)	2.68 (1.39–5.17, *p* = 0.003)*	2.69 (1.34–5.41, *p* = 0.005)*
CT or CC and smoker	86 (25.52)	48 (25.53)	1.22 (0.71–2.08, *p* = 0.479)	1.21 (0.68–2.13, *p* = 0.516)
Test for interaction	?^2^ = 11.175 (3 d.f.), *p* = 0.010*			
miRNA499 rs3746444				
TT and non-smoker	188 (55.79)	74 (39.36)	Reference	Reference
CT or CC and non-smoker	37 (10.98)	34 (18.09)	2.34 (1.36–4.00, *p* = 0.002)*	2.41 (1.36–4.26, *p* = 0.002)*
CT or CC and smoker	93 (27.60)	45 (23.94)	1.23 (0.79–1.92, *p* = 0.364)	1.37 (0.85–2.20, *p* = 0.192)
CT or CC and smoker	19 (5.64)	35 (18.62)	4.69 (2.52–8.70, *p*<0.001)*	5.64 (2.87–11.06, *p*<0.001)*
Test for interaction	?^2^ = 31.409 (3 d.f.), *p*<0.001*			

a AOR adjusted age, gender and alcohol intake.

*p value <0.05.

**Table 7:** Association of miRNA genotype and alcohol intake status.

Variable		Control	Case	OR	AOR^a^
		N = 337 (%)	N = 188 (%)	(95%CI, *p* value)	(95%CI, *p* value)
miRNA146a rs2910164					
CC and non-alcohol intake		88 (26.11)	53 (28.19)	Reference	Reference
CG or GG and non-alcohol intake		113 (33.53)	67 (35.64)	0.98 (0.62–1.55, *p* = 0.946)	0.94 (0.59–1.49, *p* = 0.792)
CG or GG and consumer		53 (15.73)	31 (16.49)	0.97 (0.56–1.70, *p* = 0.918)	0.87 (0.48–1.57, *p* = 0.651)
CG or GG and alcohol intake		83 (24.63)	37 (19.68)	0.74 (0.44–1.24, *p* = 0.253)	0.64 (0.37–1.10, *p* = 0.110)
Test for interaction		?^2^ = 1.687 (3 d.f.) *p* = 0.640			
miRNA149 rs2292832					
TT and non-alcohol intake		140 (41.54)	91 (48.40)	Reference	Reference
CT or CC and non-alcohol intake		61 (18.10)	29 (15.43)	0.73 (0.44–1.22, *p* = 0.234)	0.74 (0.44–1.26, *p* = 0.269)
CT or CC and consumer		106 (31.45)	48 (25.53)	0.70 (0.45–1.07, *p* = 0.100)	0.61 (0.38–0.97, *p* = 0.036)
CT or CC and alcohol intake		30 (8.90)	20 (10.64)	1.03 (0.55–1.92, *p* = 0.937)	0.98 (0.51–1.89, *p* = 0.955)
Test for interaction		?^2^ = 3.620 (3 d.f.) *p* = 0.306			
miRNA196 rs11614913					
TT and non-alcohol intake		64 (18.99)	38 (20.21)	Reference	Reference
CT or CC and non-alcohol intake		137 (40.65)	82 (43.62)	1.01 (0.62–1.64, *p* = 0.974)	0.95 (0.58–1.57, *p* = 0.854)
CT or CC and consumer		36 (10.68)	28 (14.89)	1.31 (0.69–2.48, *p* = 0.406)	1.05 (0.53–2.10, *p* = 0.884)
CT or CC and alcohol intake		100 (29.67)	40 (21.28)	0.67 (0.39–1.16, *p* = 0.154)	0.53 (0.29–0.96, *p* = 0.036)*
Test for interaction		?^2^ = 5.293 (3 d.f.) *p* = 0.152			
miRNA499 rs3746444					
TT and non-alcohol intake		163 (48.37)	78 (41.49)	Reference	Reference
CT or CC and non-alcohol intake		38 (11.28)	42 (22.34)	2.31 (1.38–3.87, *p* = 0.001)*	2.43 (1.43–4.12, *p* = 0.001)*
CT or CC and consumer		118 (35.01)	41 (21.81)	0.73 (0.47–1.13, *p* = 0.160)	0.66 (0.41–1.06, *p* = 0.085)
CT or CC and alcohol intake		18 (5.34)	27 (14.36)	3.14 (1.63–6.03, *p*<0.001)*	3.44 (1.69–7.00, *p*<0.001)*
Test for interaction	?^2^ = 29.345 (3 d.f.) *p*<0.001*				

a AOR adjusted age, gender and smoking status.

*p value <0.05.

## Discussion

The results of this investigation indicate that miRNA499 rs3746444 is highly associated with hepatocellular carcinoma. Regarding genotype distribution and environmental risk factors, such as smoking and alcohol consumption, miRNA499 plays a crucial role in hepatocellular carcinoma.

MiRNA499 rs3746444 has been discussed in relation to numerous cancers. A meta-analysis of 7188 patients with several cancers compared with 8548 control participants reported that a significant association exists between miR499 rs3746444 polymorphisms and an increased risk of cancer [33], [34]. MiRNA499 rs3746444 has been reported to increase susceptibility to hepatocellular carcinoma in the Chinese population [35]. The results obtained after increasing the sample size were similar. Several studies of Asian populations have reported the same finding; that is, miRNA499 is associated with cancer [36], [37]. However, in Caucasian population, miRNA499 has not been associated with cancer [38]. Our findings may through the racial difference and it’s specifically direct against to Asians. Furthermore, the basic laboratory study also demonstrated that miRNA499 may bind to and reduce the expression of c-MET mRNA, which promotes cell apoptosis and inhibits cell proliferation [39].

Previous studies have shown that miRNA146 can be used as a biomarker and therapeutic target for peripartum cardiomyopathy [40], and may negatively regulate the activity of bone marrow stem cells [41]. Other studies have linked miRNA146 to inflammatory immune system responses or T-cell activity [42], [43]. Compared to the laboratory status, we observed a significant difference in the AST/ALT ratio of patients who carried CG or GG genotypes compared with those who carried the CC genotype, which may related to the liver damage caused by alcohol. Further, serum liver enzymes were associated with long-term mortality, especially the populations with low ALT and AST levels [44].

A slight difference exists in distant tumor metastasis caused by miRNA149 rs2292832 gene polymorphisms (Table S2 in File S1). Recent studies have shown that miRNA149 gene polymorphisms are associated with the prognosis for patients diagnosed with head and neck carcinoma [45]. Øster et al., also found that miRNA149 can regulate SPRX2 expression and CpG promotes hypomethylation in colorectal cancer [46]. Several in vivo studies have associated miRNA149 with the p53 gene and reported that miRNA149 may serve as an oncogenic regulator of p53 expression [47]. We observed a slight difference in distant tumor metastasis caused by miRNA149; however, a larger sample size and additional laboratory experiments are required to verify this finding.

In this study, miRNA196 exhibited a slight association with hepatocellular carcinoma after patients’ alcohol consumption was considered. However, the test results for an interaction between gene polymorphisms and cancer risk factors were not statistically significant (*p = *0.152). Previous studies have shown that miRNA196 is associated with transcription factors and affects cancer development and progression [48], [49]. In addition, increased miRNA196 expression improves the survival and prognosis for patients diagnosed with leukemia [50]. Nonetheless, the results obtained in this research do not provide sufficient evidence of a relationship between miR196 and hepatocellular carcinoma. Furthermore, after considering environmental risk factors, such as smoking and alcohol consumption, we combined the gene polymorphisms and analysis results of the interactions caused by the 2 risk factors. Considered with the viral factors, carried miR34b/c rs4938723 CC was significantly associated with HCC. And with miR196a rs11614913 was not associated with HCC, but carried CC genotype significantly enhanced the influences caused by rs4938723 in women population [51]. For patients in the smoking and alcohol consumption groups, mirRNA499 can cause interactions (*p*<0.001) and increase the OR for developing hepatocellular carcinoma. This may be because the various functions of miRNAs not only influence tumor differentiation and proliferation, but also promote inflammation and apoptosis.

Sample size was the greatest limitation in our study. The sample comprised a fewer number of female participants than male participants, which may be due to differences in lifestyle factors, such as smoking status and alcohol consumption. Substantial exposure to the environmental risk factors can easily result in underestimation of gene affections in subsequent analyses. We used a regression model to reduce the effect of potential confounding factors, but estimating the genetic effects under environmental affections remained difficult.

In conclusion, we observed a strong relationship between miRNA499 gene polymorphisms and hepatocellular carcinoma. People who carry miRNA499 rs3746444 CT or CC genotypes may have a high susceptibility to hepatocellular carcinoma. In addition, we observed a strong interaction and increased odds ratio for hepatocellular carcinoma development between miRNA499 gene polymorphisms and smoking and alcohol consumption. Although we observed evidence that miRNA499 rs3746444 is related to hepatocellular carcinoma susceptibility, additional laboratory experiments and functional assays are required to confirm the effects of miRNA499 rs3746444 gene polymorphisms on hepatocellular carcinoma.

## Supporting Information

File S1 **Distribution of miRNAs genotypes in liver cancer patients.** Table S1. Distribution of miRNAs genotypes with Hepatitis B in healthy controls and liver cancer patients. Table S2. Relationship of clinical TNM stages and miRNAs genotypes in male liver cancer patients. Table S3. Relationship of clinical TNM stages and miRNAs genotypes in female liver cancer patients.
(DOC)

## References

[1] Zhuang L, Zeng X, Yang Z, Meng Z (2013) Effect and safety of interferon for hepatocellular carcinoma: a systematic review and meta-analysis. PLoS One 8: e61361. 24069133 10.1371/journal.pone.0061361 PMC3775819

[2] Weinmann A, Koch S, Niederle IM, Schulze-Bergkamen H, König J, et al.. (2013) Trends in Epidemiology, Treatment, and Survival of Hepatocellular Carcinoma Patients Between 1998 and 2009: An Analysis of 1066 Cases of a German HCC Registry. J Clin Gastroenterol in press. 10.1097/MCG.0b013e3182a8a793 24045276

[3] Huang YW, Wang TC, Lin SC, Chang HY, Chen DS, et al (2013) Increased risk of cirrhosis and its decompensation in chronic hepatitis B patients with new onset diabetes: A nationwide cohort study. Clin Infect Dis 57: 1695–1702. 24051864 10.1093/cid/cit603

[4] Yin J, Zhang H, He Y, Xie J, Liu S, et al (2010) Distribution and hepatocellular carcinoma-related viral properties of hepatitis B virus genotypes in Mainland China: a community-based study. Cancer Epidemiol Biomarkers Prev 19: 777–786. 20160279 10.1158/1055-9965.EPI-09-1001

[5] Zhang Q, Cao G (2011) Genotypes, mutations, and viral load of hepatitis B virus and the risk of hepatocellular carcinoma: HBV properties and hepatocarcinogenesis. Hepat Mon 11: 86–91. 22087123 PMC3206676

[6] Bartel DP (2004) MicroRNAs: Genomics, Biogenesis, Mechanism, and Function. Cell 116: 281–297. 14744438 10.1016/s0092-8674(04)00045-5

[7] Ambros V (2004) The functions of animal microRNAs. Nature 431: 350–355. 15372042 10.1038/nature02871

[8] Feng MJ, Shi F, Qiu C, Peng WK (2012) MicroRNA-181a, -146a and -146b in spleen CD4+ T lymphocytes play proinflammatory roles in a murine model of asthma. Int Immunopharmacol 13: 347–353. 22580216 10.1016/j.intimp.2012.05.001

[9] Perry MM, Baker JE, Gibeon DS, Adcock IM, Chung KF (2014) Airway Smooth Muscle Hyperproliferation is Regulated by microRNA-221 in Severe Asthma. Am J Respir Cell Mol Biol 50: 7–17. 23944957 10.1165/rcmb.2013-0067OC PMC3930931

[10] de Rinaldis E, Gazinska P, Mera A, Modrusan Z, Fedorowicz GM, et al (2013) Integrated genomic analysis of triple-negative breast cancers reveals novel microRNAs associated with clinical and molecular phenotypes and sheds light on the pathways they control. BMC Genomics 14: 643. 24059244 10.1186/1471-2164-14-643 PMC4008358

[11] Sureban SM, May R, Qu D, Weygant N, Chandrakesan P, et al (2013) DCLK1 Regulates Pluripotency and Angiogenic Factors via microRNA-Dependent Mechanisms in Pancreatic Cancer. PLoS One 8: e73940. 24040120 10.1371/journal.pone.0073940 PMC3767662

[12] Zhao G, Zhang JG, Shi Y, Qin Q, Liu Y, et al (2013) MiR-130b Is a Prognostic Marker and Inhibits Cell Proliferation and Invasion in Pancreatic Cancer through Targeting STAT3. PLoS One 8: e73803. 24040078 10.1371/journal.pone.0073803 PMC3769379

[13] Li T, Leong MH, Harms B, Kennedy G, Chen L (2013) MicroRNA-21 as a potential colon and rectal cancer biomarker. World J Gastroenterol 19: 5615–5621. 24039353 10.3748/wjg.v19.i34.5615 PMC3769897

[14] Xu L, Zhou X, Qiu MT, Yin R, Wu YQ, et al (2013) Lack of Association between Hsa-miR-149 rs2292832 Polymorphism and Cancer Risk: A Meta-Analysis of 12 Studies. PLoS One 8: e73762. 24040059 10.1371/journal.pone.0073762 PMC3764043

[15] Linhares JJ, Azevedo M Jr, Siufi AA, de Carvalho CV, Wolgien Mdel C, et al (2012) Evaluation of single nucleotide polymorphisms in microRNAs (hsa-miR-196a2 rs11614913 C/T) from Brazilian women with breast cancer. BMC Med Genet 13: 119. 23228090 10.1186/1471-2350-13-119 PMC3563578

[16] Zheng J, Deng J, Xiao M, Yang L, Zhang L, et al (2013) A Sequence Polymorphism in miR-608 Predicts Recurrence after Radiotherapy for Nasopharyngeal Carcinoma. Cancer Res 73: 5151–5162. 23796562 10.1158/0008-5472.CAN-13-0395

[17] Sabina S, Pulignani S, Rizzo M, Cresci M, Vecoli C, et al (2013) Germline hereditary, somatic mutations and microRNAs targeting-SNPs in congenital heart defects. J Mol Cell Cardiol 60: 84–89. 23583740 10.1016/j.yjmcc.2013.04.002

[18] Zhong S, Chen Z, Xu J, Li W, Zhao J (2013) Pre-mir-27a rs895819 polymorphism and cancer risk: a meta-analysis. Mol Biol Rep 40: 3181–3186. 23266669 10.1007/s11033-012-2392-3

[19] de Larrea CF, Navarro A, Tejero R, Tovar N, Díaz T, et al (2012) Impact of MiRSNPs on survival and progression in patients with multiple myeloma undergoing autologous stem cell transplantation. Clin Cancer Res 18: 3697–3704. 22539802 10.1158/1078-0432.CCR-12-0191

[20] Serrano NA, Xu C, Liu Y, Wang P, Fan W, et al (2012) Integrative analysis in oral squamous cell carcinoma reveals DNA copy number-associated miRNAs dysregulating target genes. Otolaryngol Head Neck Surg 147: 501–508. 22470160 10.1177/0194599812442490 PMC7068663

[21] Liu Z, Wei S, Ma H, Zhao M, Myers JN, et al (2011) A functional variant at the miR-184 binding site in TNFAIP2 and risk of squamous cell carcinoma of the head and neck. Carcinogenesis 32: 1668–1674. 21934093 10.1093/carcin/bgr209 PMC3204352

[22] Teo MT, Landi D, Taylor CF, Elliott F, Vaslin L, et al (2012) The role of microRNA-binding site polymorphisms in DNA repair genes as risk factors for bladder cancer and breast cancer and their impact on radiotherapy outcomes. Carcinogenesis 33: 581–586. 22166496 10.1093/carcin/bgr300 PMC3291859

[23] Liu CJ, Shen WG, Peng SY, Cheng HW, Kao SY, et al (2014) miR-134 induces oncogenicity and metastasis in head and neck carcinoma through targeting WWOX gene. Int J Cancer 134: 811–821. 23824713 10.1002/ijc.28358

[24] Rani SB, Rathod SS, Karthik S, Kaur N, Muzumdar D, et al (2013) MiR-145 functions as a tumor-suppressive RNA by targeting Sox9 and adducin 3 in human glioma cells. Neuro Oncol 15: 1302–1316. 23814265 10.1093/neuonc/not090 PMC3779040

[25] Jeon YJ, Kim OJ, Kim SY, Oh SH, Oh D, et al (2013) Association of the miR-146a, miR-149, miR-196a2, and miR-499 polymorphisms with ischemic stroke and silent brain infarction risk. Arterioscler Thromb Vasc Biol 33: 420–430. 23202363 10.1161/ATVBAHA.112.300251

[26] Boldin MP, Taganov KD, Rao DS, Yang L, Zhao JL, et al (2011) miR-146a is a significant brake on autoimmunity, myeloproliferation, and cancer in mice. J Exp Med 208: 1189–1201. 21555486 10.1084/jem.20101823 PMC3173243

[27] Wang F, Ma YL, Zhang P, Shen TY, Shi CZ, et al (2013) SP1 mediates the link between methylation of the tumour suppressor miR-149 and outcome in colorectal cancer. J Pathol 229: 12–24. 22821729 10.1002/path.4078

[28] Schotte D, Lange-Turenhout EA, Stumpel DJ, Stam RW, Buijs-Gladdines JG, et al (2010) Expression of miR-196b is not exclusively MLL-driven but is especially linked to activation of HOXA genes in pediatric acute lymphoblastic leukemia. Haematologica 95: 1675–1682. 20494936 10.3324/haematol.2010.023481 PMC2948092

[29] Liu X, Zhang Z, Sun L, Chai N, Tang S, et al (2011) MicroRNA-499–5p promotes cellular invasion and tumor metastasis in colorectal cancer by targeting FOXO4 and PDCD4. Carcinogenesis 32: 1798–1805. 21934092 10.1093/carcin/bgr213

[30] Yu YL, Su KJ, Chen CJ, Wei CW, Lin CJ, et al (2012) Synergistic anti-tumor activity of isochaihulactone and paclitaxel on human lung cancer cells. J Cell Physiol 227: 213–222. 21391217 10.1002/jcp.22719

[31] Chu YH, Tzeng SL, Lin CW, Chien MH, Chen MK, et al (2012) Impacts of microRNA gene polymorphisms on the susceptibility of environmental factors leading to carcinogenesis in oral cancer. PLoS One 7: e39777. 22761899 10.1371/journal.pone.0039777 PMC3386241

[32] Wei CW, Lin CC, Yu YL, Lin CY, Lin PC, et al (2009) n-Butylidenephthalide induced apoptosis in the A549 human lung adenocarcinoma cell line by coupled down-regulation of AP-2alpha and telomerase activity. Acta Pharmacol Sin 30: 1297–1306. 19701232 10.1038/aps.2009.124 PMC4007174

[33] Wang F, Sun G, Zou Y, Li Y, Hao L, et al (2012) Association of microRNA-499 rs3746444 polymorphism with cancer risk: evidence from 7188 cases and 8548 controls. PLoS One 7: e45042. 22970328 10.1371/journal.pone.0045042 PMC3438197

[34] Wang Y, Yang B, Ren X (2012) Hsa-miR-499 polymorphism (rs3746444) and cancer risk: a meta-analysis of 17 case-control studies. Gene 509: 267–272. 22922391 10.1016/j.gene.2012.08.008

[35] Xiang Y, Fan S, Cao J, Huang S, Zhang LP (2012) Association of the microRNA-499 variants with susceptibility to hepatocellular carcinoma in a Chinese population. Mol Biol Rep 39: 7019–7023. 22311030 10.1007/s11033-012-1532-0

[36] Qiu MT, Hu JW, Ding XX, Yang X, Zhang Z, et al (2012) Hsa-miR-499 rs3746444 polymorphism contributes to cancer risk: a meta-analysis of 12 studies. PLoS One 7: e50887. 23236400 10.1371/journal.pone.0050887 PMC3517592

[37] Chen P, Zhang J, Zhou F (2012) miR-499 rs3746444 polymorphism is associated with cancer development among Asians and related to breast cancer susceptibility. Mol Biol Rep 39: 10433–10438. 23053947 10.1007/s11033-012-1922-3

[38] Wang L, Qian S, Zhi H, Zhang Y, Wang B, et al (2012) The association between hsa-miR-499 T>C polymorphism and cancer risk: a meta-analysis. Gene 508: 9–14. 22903035 10.1016/j.gene.2012.08.005

[39] Buurman R, Gürlevik E, Schäffer V, Eilers M, Sandbothe M, et al (2012) Histone deacetylases activate hepatocyte growth factor signaling by repressing microRNA-449 in hepatocellular carcinoma cells. Gastroenterology 143: 811–820. 22641068 10.1053/j.gastro.2012.05.033

[40] Halkein J, Tabruyn SP, Ricke-Hoch M, Haghikia A, Nguyen NQ, et al (2013) MicroRNA-146a is a therapeutic target and biomarker for peripartum cardiomyopathy. J Clin Invest 123: 2143–2154. 23619365 10.1172/JCI64365 PMC3638905

[41] Matysiak M, Fortak-Michalska M, Szymanska B, Orlowski W, Jurewicz A, et al (2013) MicroRNA-146a negatively regulates the immunoregulatory activity of bone marrow stem cells by targeting prostaglandin E2 synthase-2. J Immunol 190: 5102–5109. 23589616 10.4049/jimmunol.1202397

[42] Ichii O, Otsuka S, Sasaki N, Namiki Y, Hashimoto Y, et al (2012) Altered expression of microRNA miR-146a correlates with the development of chronic renal inflammation. Kidney Int 81: 280–292. 21975861 10.1038/ki.2011.345

[43] Yang L, Boldin MP, Yu Y, Liu CS, Ea CK, et al (2012) miR-146a controls the resolution of T cell responses in mice. J Exp Med 209: 1655–1670. 22891274 10.1084/jem.20112218 PMC3428948

[44] Koehler EM, Sanna D, Hansen BE, van Rooij FJ, Heeringa J, et al (2014) Serum liver enzymes are associated with all-cause mortality in an elderly population. Liver Int 34: 296–304. 24219360 10.1111/liv.12311

[45] Tu HF, Liu CJ, Chang CL, Wang PW, Kao SY, et al (2012) The association between genetic polymorphism and the processing efficiency of miR-149 affects the prognosis of patients with head and neck squamous cell carcinoma. PLoS One 7: e51606. 23272122 10.1371/journal.pone.0051606 PMC3522737

[46] Øster B, Linnet L, Christensen LL, Thorsen K, Ongen H, et al (2013) Non-CpG island promoter hypomethylation and miR-149 regulate the expression of SRPX2 in colorectal cancer. Int J Cancer 132: 2303–2315. 23115050 10.1002/ijc.27921

[47] Jin L, Hu WL, Jiang CC, Wang JX, Han CC, et al (2011) MicroRNA-149, a p53-responsive microRNA, functions as an oncogenic regulator in human melanoma. Proc Natl Acad Sci U S A 108: 15840–15845. 21896753 10.1073/pnas.1019312108 PMC3179083

[48] Li Y, Zhang M, Chen H, Dong Z, Ganapathy V, et al (2010) Ratio of miR-196s to HOXC8 messenger RNA correlates with breast cancer cell migration and metastasis. Cancer Res 70: 7894–7904. 20736365 10.1158/0008-5472.CAN-10-1675 PMC2955846

[49] Chen C, Zhang Y, Zhang L, Weakley SM, Yao Q (2011) MicroRNA-196: critical roles and clinical applications in development and cancer. J Cell Mol Med 15: 14–23. 21091634 10.1111/j.1582-4934.2010.01219.x PMC3276076

[50] Popovic R, Riesbeck LE, Velu CS, Chaubey A, Zhang J, et al (2009) Regulation of mir-196b by MLL and its overexpression by MLL fusions contributes to immortalization. Blood 113: 3314–3322. 19188669 10.1182/blood-2008-04-154310 PMC2665896

[51] Han Y, Pu R, Han X, Zhao J, Zhang Y, et al (2013) Associations of pri-miR-34b/c and pre-miR-196a2 polymorphisms and their multiplicative interactions with hepatitis B virus mutations with hepatocellular carcinoma risk. PLoS One 8: e58564. 23516510 10.1371/journal.pone.0058564 PMC3596299

