# Supplementary material for: MicroRNA Gene Polymorphisms and Environmental Factors Increase Patient Susceptibility to Hepatocellular Carcinoma
Source: PLoS One. 2014 Feb 26;9(2):e89930. doi: 10.1371/journal.pone.0089930 (PMC3935960; doi:10.1371/journal.pone.0089930)
Supplement: File S1 — Distribution of miRNAs genotypes in liver cancer patients. Table S1. Distribution of miRNAs genotypes with Hepatitis B in healthy controls and liver cancer patients. Table S2. Relationship of clinical TNM stages and miRNAs genotypes in male liver cancer patients. Table S3. Relationship of clinical TNM stages and miRNAs genotypes in female liver cancer patients. (DOC) [file pone.0089930.s001.doc]

**File S1: Distribution of miRNAs genotypes in liver cancer patients**

| **Table S1.** Distribution of miRNAs genotypes with Hepatitis B in healthy controls and liver cancer patients. | | | | |
| --- | --- | --- | --- | --- |
| Gene | Control | Case | OR | AOR^a^ |
|  | N=34 (%) | N=80 (%) | (95% CI, *p* value) | (95% CI, *p* value) |
| miRNA146a rs2910164 |  |  |  |  |
| CC | 12 (35.29) | 32 (40.00) | Reference | Reference |
| CG | 16 (47.05) | 38 (47.50) | 0.89 (0.37-2.16, *p*=0.797) | 0.78 (0.31-1.99, *p*=0.700) |
| GG | 6 (17.64) | 10 (12.50) | 0.63 (0.19-2.10, *p*=0.447) | 0.62 (0.16-2.25, *p*=0.123) |
| CG / GG | 22 (64.71) | 48 (60.00) | 0.82 (0.36-1.88, *p*=0.637) | 0.70 (0.32-1.53, *p*=0.376) |
|  |  |  |  |  |
| miRNA149 rs2292832 |  |  |  |  |
| TT | 24 (70.59) | 58 (72.50) | Reference | Reference |
| CT | 8 (23.53) | 16 (20.00) | 0.83 (0.31-2.19, *p*=0.703) | 0.86 (0.32-2.35, *p*=0.676) |
| CC | 2 (5.88) | 6 (7.50) | 1.24 (0.23-6.59, *p*=0.800) | 1.22 (0.19-7.66, *p*=0.833) |
| CT / CC | 10 (29.41) | 22 (27.50) | 0.91 (0.38-2.21, *p*=0.835) | 1.02 (0.42-2.50, *p*=0.966) |
|  |  |  |  |  |
| miRNA196 rs11614913 |  |  |  |  |
| TT | 7 (20.59) | 33 (41.25) | Reference | Reference |
| CT | 17 (50.00) | 31 (38.75) | 0.39 (0.14-1.06, *p*=0.064) | 0.37 (0.13-1.11, *p*=0.076) |
| CC | 10 (29.41) | 16 (20.00) | 0.34 (0.11-1.06, *p*=0.062) | 0.36 (0.11-1.19, *p*=0.093) |
| CT / CC | 27 (79.41) | 47 (58.75) | 0.37 (0.14-0.95, *p*=0.038)* | 0.53 (0.24-0.13, *p*=0.039)* |
|  |  |  |  |  |
| miRNA499 rs3746444 |  |  |  |  |
| TT | 31 (91.18) | 54 (67.5) | Reference | Reference |
| CT | 2 (5.88) | 22 (27.5) | 6.32 (1.39-28.68, *p*=0.017)* | 6.14 (1.32-28.56, *p*=0.020)* |
| CC | 1 (2.94) | 4 (5.00) | 2.23 (1.25-21.47, *p*=0.016)* | 3.02 (0.30-30.93, *p*=0.351) |
| CT / CC | 3 (8.82) | 26 (32.5) | 4.98 (1.39-17.79, *p*=0.013)* | 4.75 (1.32-17.01, *p*=0.018)* |

^a^AOR adjusted age, sex, smoking status and drinking status.

* *p* value < 0.05

| **Table S2.** Relationship of clinical TNM stages and miRNAs genotypes in male liver cancer patients. | | | | | | | | | | | | |
| --- | --- | --- | --- | --- | --- | --- | --- | --- | --- | --- | --- | --- |
| Variables | rs2910164 |  |  | rs2292832 |  |  | rs11614913 |  |  | rs3746444 |  |  |
|  | CC | CG or GG | *p* value | TT | CT or CC | *p* value | TT | CT or CC | *p* value | TT | CT or CC | *p* value |
|  | N=65 (%) | N=71 (%) |  | N=99 (%) | N=37 (%) |  | N=55 (%) | N=81 (%) |  | N=86 (%) | N=50 (%) |  |
| Clinical stage |  |  |  |  |  |  |  |  |  |  |  |  |
| Stage I/II | 35 (53.85) | 42 (59.15) | 0.533 | 57 (57.58) | 20 (54.05) | 0.712 | 33 (60.00) | 44 (54.32) | 0.512 | 50 (58.14) | 27 (54.00) | 0.639 |
| Stage III/IV | 30 (46.15) | 29 (40.85) |  | 42 (42.42) | 17 (45.95) |  | 22 (40.00) | 37 (45.68) |  | 36 (41.86) | 23 (46.00) |  |
|  |  |  |  |  |  |  |  |  |  |  |  |  |
| Tumor size |  |  |  |  |  |  |  |  |  |  |  |  |
| T1 + T2 | 35 (53.85) | 42 (59.15) | 0.533 | 58 (58.59) | 19 (51.35) | 0.449 | 32 (58.18) | 45 (55.56) | 0.762 | 50 (58.14) | 27 (54.00) | 0.639 |
| T3 + T4 | 30 (46.15) | 29 (40.85) |  | 41 (41.41) | 18 (48.65) |  | 23 (41.82) | 36 (44.44) |  | 36 (41.86) | 23 (46.00) |  |
|  |  |  |  |  |  |  |  |  |  |  |  |  |
| Lymph node metastasis | |  |  |  |  |  |  |  |  |  |  |  |
| Negative | 61 (93.85) | 68 (95.77) | 0.709 ^a^ | 93 (93.94) | 36 (97.30) | 0.673 ^a^ | 51 (92.73) | 78 (96.30) | 0.440 ^a^ | 81 (94.19) | 48 (96.00) | 1.000 ^a^ |
| Positive | 4 (6.15) | 3 (4.23) |  | 6 (6.06) | 1 (2.70) |  | 4 (7.27) | 3 (3.70) |  | 5 (5.81) | 2 (4.00) |  |
|  |  |  |  |  |  |  |  |  |  |  |  |  |
| Distant metastasis | |  |  |  |  |  |  |  |  |  |  |  |
| Negative | 59 (90.77) | 68 (95.77) | 0.310 ^a^ | 95 (95.96) | 32 (86.49) | 0.048 ^a^* | 51 (92.73) | 76 (93.83) | 1.000 ^a^ | 79 (91.86) | 48 (96.00) | 0.485 ^a^ |
| Positive | 6 (9.23) | 3 (4.23) |  | 4 (4.04) | 5 (13.51) |  | 4 (7.27) | 5 (6.17) |  | 7 (8.14) | 2 (4.00) |  |
|  |  |  |  |  |  |  |  |  |  |  |  |  |

a. Use Fisher’s exact test; * *p* value < 0.05

| **Table S3.** Relationship of clinical TNM stages and miRNAs genotypes in female liver cancer patients. | | | | | | | | | | | | |
| --- | --- | --- | --- | --- | --- | --- | --- | --- | --- | --- | --- | --- |
| Variables | rs2910164 |  |  | rs2292832 |  |  | rs11614913 |  |  | rs3746444 |  |  |
|  | CC | CG or GG | *p* value | TT | CT or CC | *p* value | TT | CT or CC | *p* value | TT | CT or CC | *p* value |
|  | N=19 (%) | N=33 (%) |  | N=40 (%) | N=12 (%) |  | N=11 (%) | N=41 (%) |  | N=33 (%) | N=19 (%) |  |
| Clinical stage |  |  |  |  |  |  |  |  |  |  |  |  |
| Stage I/II | 14 (73.68) | 24 (72.73) | 0.940 | 28 (70.00) | 10 (83.33) | 0.475 ^a^ | 7 (63.64) | 31 (75.61) | 0.460 ^a^ | 23 (69.70) | 15 (78.95) | 0.534 ^a^ |
| Stage III/IV | 5 (26.32) | 9 (27.27) |  | 12 (30.00) | 2 (16.67) |  | 4 (36.36) | 10 (24.39) |  | 10 (30.30) | 4 (21.05) |  |
|  |  |  |  |  |  |  |  |  |  |  |  |  |
| Tumor size |  |  |  |  |  |  |  |  |  |  |  |  |
| T1 + T2 | 15 (78.95) | 25 (75.76) | 1.000 ^a^ | 30 (75.00) | 10 (83.33) | 0.709 ^a^ | 7 (63.64) | 33 (80.49) | 0.253 ^a^ | 25 (75.76) | 15 (78.95) | 1.000 ^a^ |
| T3 + T4 | 4 (21.05) | 8 (24.24) |  | 10 (25.00) | 2 (16.67) |  | 4 (36.36) | 8 (19.51) |  | 8 (24.24) | 4 (21.05) |  |
|  |  |  |  |  |  |  |  |  |  |  |  |  |
| Lymph node metastasis | |  |  |  |  |  |  |  |  |  |  |  |
| Negative | 18 (94.74) | 32 (96.97) | 1.000 ^a^ | 38 (95.00) | 12 (100.0) | 1.000 ^a^ | 11 (100.0) | 39 (95.12) | 1.000 ^a^ | 31 (93.94) | 19 (19.00) | 0.527 ^a^ |
| Positive | 1 (5.26) | 1 (3.03) |  | 2 (5.00) | 0 (0.00) |  | 0 (0.00) | 2 (4.88) |  | 2 (6.06) | 0 (0.00) |  |
|  |  |  |  |  |  |  |  |  |  |  |  |  |
| Distant metastasis | |  |  |  |  |  |  |  |  |  |  |  |
| Negative | 19 (100.0) | 31 (93.94) | 0.527 ^a^ | 38 (95.00) | 12 (100.0) | 1.000 ^a^ | 11 (100.0) | 39 (95.12) | 1.000 ^a^ | 31 (93.94) | 19 (19.00) | 0.527 ^a^ |
| Positive | 0 (0.00) | 2 (6.06) |  | 2 (5.00) | 0 (0.00) |  | 0 (0.00) | 2 (4.88) |  | 2 (6.06) | 0 (0.00) |  |
|  |  |  |  |  |  |  |  |  |  |  |  |  |

a. Use Fisher’s exact test; * *p* value < 0.05
